# Toddlers’ Ability to Leverage Statistical Information to Support Word Learning

**DOI:** 10.3389/fpsyg.2021.600694

**Published:** 2021-04-09

**Authors:** Erica M. Ellis, Arielle Borovsky, Jeffrey L. Elman, Julia L. Evans

**Affiliations:** ^1^Department of Communication Disorders, California State University, Los Angeles, Los Angeles, CA, United States; ^2^Department of Speech, Language, and Hearing Sciences, Purdue University, West Lafayette, IN, United States; ^3^Center for Research in Language, University of California, San Diego, La Jolla, CA, United States; ^4^Department of Cognitive Science, University of California, San Diego, La Jolla, CA, United States; ^5^School of Behavioral and Brain Sciences, The University of Texas at Dallas, Richardson, TX, United States

**Keywords:** word learning, statistical word segmentation, vocabulary, individual differences, language development

## Abstract

**Purpose:**

This study investigated whether the ability to utilize statistical regularities from fluent speech and map potential words to meaning at 18-months predicts vocabulary at 18- and again at 24-months.

**Method:**

Eighteen-month-olds (*N* = 47) were exposed to an artificial language with statistical regularities within the speech stream, then participated in an object-label learning task. Learning was measured using a modified looking-while-listening eye-tracking design. Parents completed vocabulary questionnaires when their child was 18-and 24-months old.

**Results:**

Ability to learn the object-label pairing for words after exposure to the artificial language predicted productive vocabulary at 24-months and amount of vocabulary change from 18- to 24 months, independent of non-verbal cognitive ability, socio-economic status (SES) and/or object-label association performance.

**Conclusion:**

Eighteen-month-olds’ ability to use statistical information derived from fluent speech to identify words within the stream of speech and then to map the “words” to meaning directly predicts vocabulary size at 24-months and vocabulary change from 18 to 24 months. The findings support the hypothesis that statistical word segmentation is one of the important aspects of word learning and vocabulary acquisition in toddlers.

## Introduction

The course of typical vocabulary development varies tremendously between children (Fenson et al., 1994). For example, by 12 months some children may only understand 20 words, while others understand more than 150, and this variability in receptive vocabulary persists and is echoed in productive vocabulary skills throughout toddlerhood. What accounts for these initial and ongoing differences in early lexical development? We explore the possibility that variability in speech segmentation skills may underlie these ongoing differences in early word acquisition. Before infants can successfully link appropriate sound sequences to their referents, they must determine the set of sounds within a speech stream that correspond to potential words. For example, the infant must segment the auditory stimulus “*that is such a cute doggie*” into meaningful chunks to accurately link it to its referent (e.g., “doggie”). This ability to segment speech sounds into word-level units, termed “word segmentation,” is a critical part of the word learning process (Saffran and Kirkham, 2018).

Successful word segmentation requires exposure to the patterns and probabilities of sound sequences, maintaining phonological working memory and the order of the sequence of phonemes within the stream of speech to track the transitional probability so that one can increasingly identify potential word boundaries. Infants, children, and adults are all skilled at statistical word segmentation, often termed “statistical learning skills” (Aslin and Newport, 2012; Aslin, 2014; Saffran and Kirkham, 2018). Statistical learning is the implicit ability to track regularities in linguistic or other input (visual or motor, for example) and learn from the distributional information (Saffran, 2001; Lany and Saffran, 2013). The foundational statistical learning experiments in 8-month-olds demonstrated that young infants could segment speech into potential word units using transitional probabilities or co-occurring probability information between syllables (Saffran et al., 1996; Aslin et al., 1998). Researchers have argued that the ability to learn from the patterns in the input plays an important role in language learning (i.e., phonology, grammar, lexicon; Saffran, 2001, 2003). These studies suggest that during their first year of language learning, before children begin to produce words, they start learning the patterns of the language they hear, tracking the sound combinations that correspond to potential words.

Statistical word segmentation has been demonstrated in a variety of tasks and populations, however, its long-term impact on language learning is less understood (Erickson and Thiessen, 2015). There is some supportive – though not always direct – evidence that statistical learning skills support long-range outcomes in language learning. First, a number of correlational studies in adults and children find positive relations between performance on various statistical learning tasks and language skills (Evans et al., 2009; Conway et al., 2010; Misyak and Christiansen, 2012; Kidd and Arciuli, 2016; Lany et al., 2018).

Second, there are also concurrent connections between statistical word segmentation skills and word learning in infants. Graf Estes et al. (2007) compared infants’ ability to link a novel object with labels that had been previously heard as either high or low transition-probability sound sequence labels from an artificial language (Graf Estes et al., 2007). They found that brief exposure to novel high (but not low) transitional probability sound sequences within an artificial language supported subsequent linking of these same sound sequences to objects. These findings demonstrated a crucial direct link between statistical word segmentation skills and word learning at a single age. More recently, researchers found that 20-month-old English-learning toddlers with small vocabularies were able to learn high transitional probability words in Italian, whereas infants with larger vocabularies did not (Shoaib et al., 2018). These findings suggest that sensitivity to transitional probability statistics are important for identifying potential word candidates at this age. However, individual differences in toddlers’ ability to attune selectively to the particular statistics of sound sequences in their language may contribute significantly to the course of lexical development (Shoaib et al., 2018).

Previous findings suggest a potentially important relation between toddler’s ability to statistically segment the speech stream to identify potential word candidates, and a relation with concurrent vocabulary. However, there is a critical need for studies that demonstrate a longitudinal link among early statistical learning skills and later language learning outcomes to demonstrate (both theoretically and clinically) whether and how early statistical learning skills impact language learning over time. Thus, a longitudinal relation provides insight into whether and how utilizing these statistical skills are important for longer-term language development outcomes. One of the few published reports examining the link between word segmentation and later language development involved a retrospective study in 7.5-month-olds, using the infant head-turn preference procedure (HPP) and vocabulary at 2 years of age. Researchers found a positive correlation between vocabulary size at 2 years and segmentation tasks at 7.5-months (Newman et al., 2006). Singh et al. (2012) conducted a prospective study using HPP measures of word segmentation with 7.5-month-olds. Infants were familiarized with isolated words, and then tested to see how they recognized those words in a fluent speech stream. Researchers found a strong association between word segmentation abilities at 7.5-months of age and productive vocabulary size as well as general cognitive scores at 2 years of age. These findings support the validity of word segmentation using HPP tasks and suggest the association between word segmentation with later vocabulary skills outcomes. However, neither Newman et al. (2006) nor Singh et al. (2012) directly assessed whether segmentation skills directly supported word learning, or if some other mediating factor supports these longer-term correlations.

Research has emphasized that early language learners have the ability to use statistical information to segment speech in a wide variety of conditions (Krogh et al., 2013; Saffran and Kirkham, 2018), yet, there is a paucity of research that examines precisely how, or if, variability in these skills supports longer-range language outcomes. Our study seeks to address these questions by measuring how young children use statistical information to support learning of individual words and how using the information relates to current and later language outcomes as demonstrated in productive vocabulary skills.

The purpose of the present study was to examine the variability in how toddlers utilize statistical information after exposure to an artificial language and the relation to productive vocabulary at 18 and 24 months. Based on this prior evidence (reviewed above) that demonstrated separate connections between statistical learning (word segmentation), word learning and vocabulary outcomes, we predicted our data should demonstrate two primary patterns: (1) first, we expected to find toddlers are better at learning words that have been previously heard in high-transitional probability sequences, consistent with Graf Estes et al. (2007) and (2) second, we predicted toddlers’ abilities to learn high-transitional probability words should relate to their concurrent and later vocabulary at 18 and 24 months of age. Alternatively, if leveraging statistical information to identify potential word candidates is not a key mechanism in vocabulary acquisition at this stage of language development, then we should not expect to see benefits from the exposure to high transitional probability speech segments prior to word learning, or to vocabulary skills during this age range.

Importantly, the study design included a crucial control: we measured word learning for words that were either (1) Statistically exposed in high transitional probability sound sequences, or (2) Not statistically exposed. In this way, we could determine whether potential ties to current and later vocabulary outcomes were simply a relation of general learning skills (if positively associated with both 1 and 2), or to using statistical information to support learning, specifically (if positively associated with just 1). Thus, our study uniquely addresses an important gap in the literature tying together how leveraging statistical information may influence word learning and overall vocabulary outcomes.

## Materials and Methods

### Participants

Fifty-four 18-month-olds (*M* = 18 months 14 days, SD = 12.72 days; 28 female and 26 male) from monolingual English-speaking homes participated in the current study. All families resided in the surrounding metropolitan region of San Diego, California. Toddler participants were reported by parents to have normal medical histories, hearing, vision, and no known neurological impairments or developmental disabilities. All mothers reported a near-term or full-term pregnancy. During the lab visit, each participant’s middle ear function was assessed using tympanometry, and parents confirmed newborn hearing screening results. An additional eight participants began the study, but ultimately were excluded from all analyses – three for not completing the experimental tasks (crying; fussiness), three for participating only for the first visit and then dropping from the study, one for possible hearing difficulties, and one for low cognitive scores.

Overall, the sample was consistent with the demographic representation of the San Diego area. Caucasian participants represented 72% of the sample (of which 14% were reported to be Hispanic), 5% reported a Black/African-American background, 4% identified as Asian or other, and 19% identified having two or more races (retrieved from United States Census, 2010). Mothers of the toddlers were on average 33.75 (SD = 4.93) years of age. We used years of maternal education (*M* = 16.55, SD = 1.57) as an indicator of socioeconomic status (SES). All mothers had completed at least a high school degree, and a majority had completed a college degree. Thirty-seven percent of mothers worked full-time, 23% worked part-time, and 40% stayed at home or were on leave. Eighty-eight percent of fathers worked full-time, 4% worked part-time, and 8% stayed at home.

## Procedure

Parents interested in participating in the study responded to flyers about a UCSD IRB approved research project exploring lexical, semantic, and cognitive processing in toddlers. Interested parents contacted the research lab and completed a brief phone screening to determine eligibility. Eligible families were mailed a packet containing information regarding appointment date, location, and a vocabulary questionnaire (*MacArthur Bates Communicative Development Inventory: Words* and *Sentences; MBCDI-WS*, Fenson et al., 2007) to be completed prior to the laboratory appointment.

### Time One: 18-Month Visit

Each toddler was seen individually for standardized language and cognitive development assessments, as well as for the experimental tasks at 18 months. Cognitive abilities were assessed using the Cognitive Scale of the Bayley Scales of Infant Development (BSID-III) (Bayley, 2005), which allowed us to confirm our sample included children within the normal range of cognitive abilities as we examined the variability of their language skills. Therefore, we excluded participants who had cognitive skills outside of the normal range. For all subsequent analysis, we only included data from participants (*N* = 47) with scores that fell within one standard deviation of the mean on the cognitive scales of the BSID-III (*M* = 100.32, SD = 8.36, range = 85–115).

### Time Two: 24-Month Assessment

At 24 months of age, participants’ families were contacted again. Families received a mailed packet that included a vocabulary questionnaire (MBCDI-WS, Fenson et al., 2007) to be completed and returned to the lab.

### General Procedure

**FIGURE 1:**
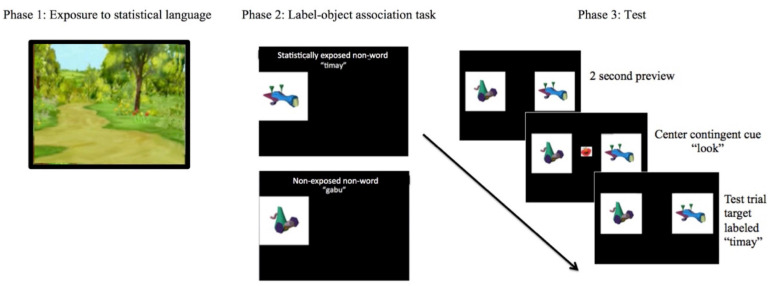
Example of task (phase 1–3).

Each toddler participant sat in their parent’s lap or in a booster seat approximately 24 inches from a 17” LCD display monitor as they viewed the task. To prevent parents from influencing their toddlers’ responses, the parents wore noise-canceling headphones during the tasks. We used a design similar Graf Estes et al. (2007) with modifications outlined below. The phases of the task were: (1) language exposure; (2) object-label association task; and (3) test phase (see Figure 1):

(1) Language exposure. Participants listened to an artificial language while sitting on their parent’s lap or booster seat and watching a silent cartoon. Stimuli were the same as used in Graf Estes et al. (2007), specifically, a recording of a trained speaker that read a sequence of syllables (approximately 20 syllables). Syllables were formed into a fluent speech stream (e.g., pigatimaydobu) that included a consistent rate (96 syllables/min) and pitch (F0 = 179 Hz) and did not include any pauses. Across the language stream, the only cue to the word boundaries was the statistical structure of the language. Specifically, the words within the language had higher transitional probabilities (1.0) while the across-word probabilities were lower (0.33) and non-words had a probability of zero. Therefore, the differences between the probabilities help contribute to signal to the listener where potential word boundaries are in the language. The syllables in this speech stream consisted of four bi-syllabic “words” (See Table 1). In a pseudorandom order, the 2.5-min stream of speech included 60 repetitions of each of the four words with no words repeated twice in a row.

**TABLE 1:** List of stimuli.

**Language exposure (Phase 1)**	**Trained Words (Phase 2)**
Language A- novel words piga dobu timay mano	timay (exposed word) gabu (non-word)
Language B- novel words pido gabu mati nomay	nomay (exposed word) dobu (non-word)

*Participants were exposed to either language A or language B.*

(2) Object-label association task. Rather than using the habituation task as in prior work by Graf Estes et al. (2007), we used a fixed duration, explicit object-label mapping task in which the number of object-labeling repetitions was identical for all children. This choice was motivated by previous pilot work which revealed that the traditional habituation criterion methods were not sensitive to learning patterns in toddlers with very low vocabulary skills (Ellis et al., 2010). In this pilot work, we found quantitative and qualitative differences in the pattern of novel word learning for low vocabulary children as the group required double the exposure as the control group before showing the novelty preference. Therefore, this fixed exposure allowed us to better explore the relationship between statistical word segmentation in young toddlers with a wide range of vocabulary abilities, rather than a habituation criterion where the number of trials would vary by participant. While toddler attention during training was not explicitly controlled, we could control the “opportunity” to attend to the label-object pair for a particular number of trials and exposures of the target word for each participant. Our goal was to control the number of exposures to allow for equal opportunity to learn the pairs, whereas in Graf Estes et al. (2007), some children may have had as many as 25 trials of exposure or as few as five trials. We monitored the training phase to see that toddlers were looking toward the screen at the images and noted any major deviations of attention.

During the object-label association task, the toddlers saw the two object-label pairs (one novel word that was previously exposed as a high-transitional probability segment in the artificial language, and one non-word that was not previously exposed) presented one at a time for 20 s. Each pair was trained across two of four trials, which occurred immediately after the language exposure phase. During the explicit object-label trials, the novel object moved back and forth across the screen while the target label was repeated seven times (therefore each object was labeled a total of 14 times across two 20 s blocks). Importantly, while the methods are slightly different than the Graf Estes et al. (2007) study, we are measuring similar skills of object-label linking.

### Experimental Task Stimuli

#### Auditory Stimuli

Participants had the opportunity to learn two pairings between the two novel objects and two word labels. Novel labels were pronounceable combinations of syllables (two syllables with low phonotactic frequency, but high transitional probability) from Language 1 (e.g., *timay*) or Language 2 (e.g., *gabu*). One of the two labels was a previously statistically *exposed* high transitional probability word from the artificial statistical language the participant heard during the exposure phase (e.g., Language 1: *timay*). The second novel label was a *non-exposed* non-word from a second language (e.g., Language 2: *gabu*) the participant did not hear during the exposure phase (see Table 1). The novel labels in each language have the same phonotactic frequencies and each version of the language contains the same syllables in a different arrangement. Each participant was exposed to two of the four label options. To control for arbitrary object-label preferences, language of exposure was counterbalanced across participants to control for frequency of exposure of the syllables and possible biases in preference to particular novel words. Parents completed a checklist to verify that the participants had not previously heard the novel labels used in the study prior to the task.

#### Visual Stimuli

Two novel object pictures that were unique, three-dimensional, bright and colorful, but differed in form and color were selected and adapted versions of objects from the Fribble image database (retrieved from^1^; Tarr, 2011). These image files were edited to fit a 400 × 400 pixel square. Appearance of novel objects were counterbalanced to appear equally often at both locations on the screen (on the left or right). Additionally, each novel object served equally as often as the target and distractor across participants, and target side was counterbalanced across trials.

(3) Test phase. The test phase began immediately upon completion of the object-label association phase. Each test trial consisted of a preview and a test component. During the preview, participants saw the two novel object images on the screen (for 2 s); after the preview, a gaze-contingent attention-getter picture appeared in the center of the two pictures and participants heard “*Look*.” Once the toddler looked at the center attention-getter picture for 100 milliseconds, the picture disappeared. This adaptation was included so each participant’s eye gaze started the test trial at the same center location point. After the center picture disappeared, participants heard the target label as they simultaneously continued to see the images of both objects. Six test trials with the label of the previously exposed “word” from the artificial language and six trials included the label of a non-word, for a total of 12 test trials. Looking behavior to: (1) the word; and (2) the non-word was measured for later analysis. Each trial in the test phase lasted 4 s.

### Eye Movement Recording

Toddlers’ eye movements were measured in response to the labeled novel objects to explore toddlers’ moment-by-moment lexical processing of the novel word meanings. Eye movements were recorded at 500 Hz using an EyeLink 2000 Remote Eyetracker (SR Research, 2011) with remote arm configuration. For each participant, the display and eye-tracking camera were placed approximately 580–620 mm from the face. Before the start of the experimental stimuli, each participant completed a brief (<2 min) five-point calibration and validation routine, to ensure that the eye-tracker was accurately calculating location of eye movements for each child.

After the initial calibration, experimental stimuli were presented using a PC computer running SR Research EyeLink Experiment Builder software (SR Research, 2011). A simple visual stimulus (i.e., bulls-eye) appeared in the center of the screen to serve as a drift check before each trial. Once the participants fixated on this center location (as verified by the EyeLink software that eye gaze was centered on the screen), the experimenter began the trial. Fixations were automatically recorded every 2 milliseconds for each trial from the onset of the images until the end of the task. The EyeLink system automatically classifies movements as saccades, fixations, and blinks using the eye-tracker’s default threshold set. Offline, the data were binned into 10 millisecond intervals, and then analyses were performed based on predefined regions of interest (1- target, 2- distractor, 3- other).

## Results

### Approach to Analysis: Eye-Tracked Data

Our data consisted of recordings of participants’ eye gaze toward images after they had been exposed to an artificial statistical language and explicitly trained to two novel object-label pairs. We used the time window of 500–2,500 milliseconds post label onset for our analyses. Research suggests the minimum latency to initiate an eye movement in infants and toddlers is approximately 233–367 milliseconds, with mean latencies considerably longer (e.g., Canfield et al., 1997). While many studies have used the time window from 300 to 1,800 milliseconds post label onset to examine lexical recognition of *familiar* words in young toddlers (Fernald et al., 1998), many *novel* word learning studies use later and longer time windows for responses than used in familiar word recognition studies (see Bion et al., 2013; Ellis et al., 2015; Borovsky et al., 2016a,b; for further discussion).

Gaze data were cleaned at the trial level. Specifically, participants were required to look to the center attention-getter for at least 100 milliseconds. While attention was allowed to vary in location across the trials, each participant needed to demonstrate looking behavior across the target and distractor trials for at least 50% of the test window for data to be used for subsequent analyses^2^. Data was then examined to determine whether looking measures were related to vocabulary outcome data. We used regression analyses to explore whether looking differences predicted current and future vocabulary outcomes.

For eye-tracking analyses, we measured proportion of looking fixations to the target “word” and then compared proportion of looking fixations to the two different object-label pairs (“word” and the other “non-word”) as a looking difference score as our main eye tracking measure of interest. We hypothesized that toddlers who demonstrate learning and utilized the statistics during the language exposure phase should indicate a greater and more positive difference score. For example, toddlers who benefit from prior exposure to the statistical language may demonstrate greater differences in proportion of looking between the word object-label pair and non-word object pair. After these initial analyses, we then examined individual differences of looking performance and examined whether learning of words varied as a function of individual differences in vocabulary size.

During the test phase, we measured the proportion of looking fixations to two different label-object pairs: (a) word (the statistically exposed high transitional probability label); and (b) the non-word (the non-exposed label). As used in previous research (Fernald et al., 2008), the proportion of looking was calculated using the looking to the target object-label pair and dividing by the total combined looking duration. The proportion of looking to word object-label during the test phase averaged 51.55%, with a wide range of performance, 21.43–79.44%. The proportion of looking to the non-word object-label pair at test averaged 48.44% also with a widespread range of looking behavior, 20.55–78.56% (see Table 2). It is important to highlight the sample’s proportion of looking data for both object-label types varied widely and, while some children clearly mapped the appropriate label to the referent, as a group, toddlers did not exhibit above chance looking in either condition. A paired samples *t*-test revealed no significant difference between the proportion of looking to the word and non-word pairs, *t*(46) = 0.739, *p* = 0.464.

**TABLE 2:** Descriptive statistics for eye-tracking measures, *N* = 47.

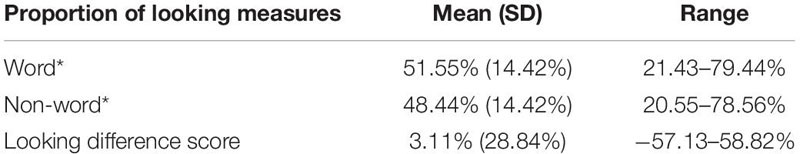

*Measures of proportion of looking to the target word or the non-word divided by total looking to target and distractor.

### Difference Scores

Despite the lack of difference in looking at the word types as a group, our primary interest was whether and to what degree looking behaviors might vary between word types at the individual level, and how these differences in learning would forecast later language learning. Therefore, we calculated the *difference* in proportion of looking between the word and non-word object-label pairs for each participant (i.e., proportion looking to word pair minus proportion looking to non-word pair) to determine whether and to what degree the exposure to the language supported learning at the individual level. For example, toddlers having difference scores with a greater proportion of looking toward target word pair were interpreted as having utilized statistical information from the previous exposure to the artificial language (see Table 2 below).

**FIGURE 2:**
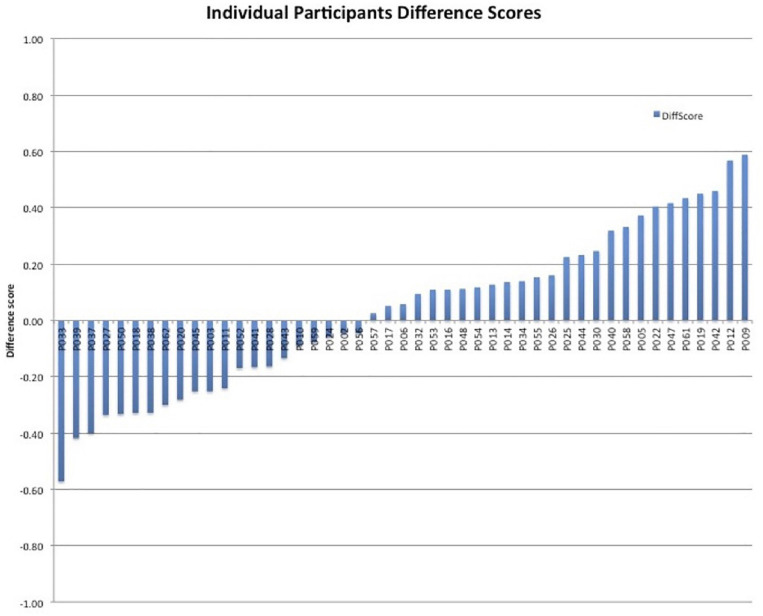
Individual participant differences scores.

By examining the *differences* in looking between the two types of object-label pairs, we were able to measure whether participants may have used the exposure to the statistics from the language prior to the object-label association and test phases or if the object-label association training phase alone was sufficient to learn the words. Although the mean difference in looking score was only 3.11% (SD = 28.84%) for the entire sample, there were tremendous individual differences (ranging from −57 to 59%), reflecting a wide range in whether and how 18-month-olds leverage their statistical segmentation skills to support word learning. Therefore, we next asked whether individual differences in statistically mediated word learning performance related to current and subsequent differences in vocabulary size and change. Figure 2 displays the individual differences among the participants by their difference score outcome.

### Vocabulary Questionnaire Data

**FIGURE 3:**
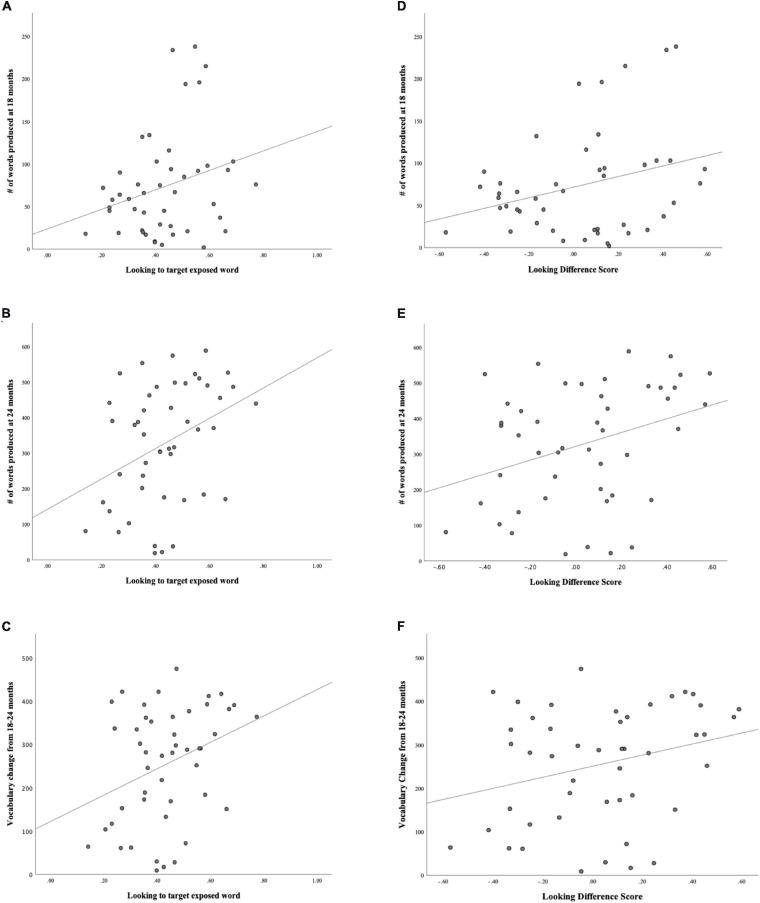
**(A)** Scatterplot of Looking to statistically exposed word and # words produced at 18 mos. (*N* = 47). **(B)** Scatterplot of Looking to statistically exposed word and # words produced at 24 mos. (*N* = 47). **(C)** Scatterplot of Looking to statistically exposed word and vocabulary change from 18-24 mos. (*N* = 47). **(D)** Scatterplot of Looking Difference score and # words at 18 mos. (*N* = 47). **(E)** Scatterplot of Looking Difference score and # words at 24 mos. (*N* = 47). **(F)** Scatterplot of Looking Difference score and Vocabulary change from 18-24 mos. (*N* = 47).

We collected vocabulary data at two separate time points (18 and 24 months). Parents completed vocabulary questionnaires (MBCDI: WS; Fenson et al., 2007) and examiners were blind to vocabulary data until after testing. A measure of total number of words produced (i.e., raw number of words) and a percentile score for words produced at each age were calculated. We also examined the total words produced by each toddler at 18 and 24 months as a direct measure of the *vocabulary change* between the two time points. For example, a toddler with 50 words at 18 months old (time 1) and 200 words at 24 months old (time 2) would have a vocabulary change of 150 words. This measure has been used in previous research studies to predict and explain language outcomes in young children (Fenson et al., 1994; Hoff, 2006; Rowe et al., 2012). The sample had an average of 73 words (37th percentile) in their productive vocabularies at 18 months, 328 words (55th percentile) at 24 months and an average vocabulary change of 255 words. The vocabulary means and standard deviations are reported in Table 3 below. Scatterplots of vocabulary and looking behaviors are displayed in Figures 3A–F.

**TABLE 3:** Descriptive statistics for vocabulary measures^a^.

**Vocabulary Measures (*N* = 47)**	**18-month Vocabulary M (SD, range)**	**24-month Vocabulary M (SD, range)**
Percentile- words produced	37.15 (SD = 26.22, range 1–93)	55.17 (SD = 29.22, range 1–96)
Number of words produced	73.17 (SD = 67.17, range 0–271)	328.13 (SD = 167.33, range 19–589)
Vocabulary change	NA	254.96 (SD = 132.46, range 9–475)

*^*a*^MacArthur-Bates Communicative Development Inventory – Words and Sentences (Fenson et al., 2007).*

### Predictors of Vocabulary

Given the range of performance during the task, we carried out exploratory analyses that measured correlations between looking to the statistically exposed “word” pairs, looking difference scores, cognitive abilities, maternal education and vocabulary measures. The correlations between the gaze measures and the cognitive and maternal education measures were non-significant. The correlations between looking to statistically exposed “word” pairs, looking difference scores and vocabulary measures were significant. The analyses revealed a significant positive relation between looking to statistically exposed “word” pairs and productive vocabulary at 18-months (*r* = 0.257, *p* = 0.041), 24-months (*r* = 0.362, *p* = 0.006) and vocabulary change between 18 and 24 months (*r* = 0.327, *p* = 0.012). The looking difference measure and productive vocabulary at 18 months (*r* = 0.287, *p* = 0.025), 24-months (*r* = 0.335, *p* = 0.011) and vocabulary change between 18 and 24 months (*r* = 0.277, *p* = 0.03) were also significant positive relations (see Table 4).

**TABLE 4:** Correlations between looking measures, cognitive abilities, maternal education (SES), and vocabulary measures.

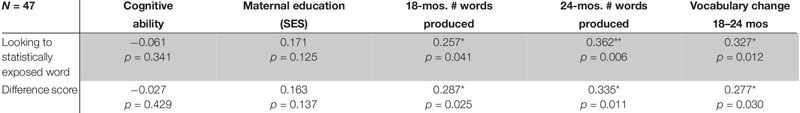

*Looking to statistically exposed word raw data were used in the analyses rather than proportion data. ^∗^<0.05 level (one-tailed). ^∗∗^<0.01 level (one-tailed).*

Next, separate multiple regression analyses were conducted, three to test the predictor of looking to the statistically exposed word and three to test the looking difference score as the predictor variable. The analyses were used to investigate how vocabulary at each age (18- and 24 months) and vocabulary change from 18–24 months may be related to word learning performance as demonstrated by looking measures at 18 months, while controlling for cognitive ability (as indicated by BSID-III Cognitive scores; Bayley, 2005), and socioeconomic status (SES, as indicated by years of maternal education). For each analysis, the independent or predictor variables of cognitive ability, SES and object-label association skill (as indicated by looking behavior to non-words) were entered first, as control variables. We next entered the measure of either looking to the statistically exposed word or the difference scores as an indication of leveraging statistical information for word learning as our final predictor.

The first regression model (Table 5A) included the control variables of cognitive skill, SES and simple object-label association did not significantly predict vocabulary at 18 months. After controlling for these variables, the relation between looking to the statistically exposed “word” pairs and vocabulary at 18 months was not significant, β = 0.227, *t*(42) = 1.28, *p* = 0.205, Cohen’s *d* = 0.39. In the second regression (Table 5B), the control variables again did not reach significance as predictors of vocabulary at 24 months. However, the toddlers’ looking to the statistically exposed “word” pairs performance in the experimental task accounted for a significant amount of variance in their vocabulary at 24 months with a moderate-to-large effect size, independent of cognitive skill, SES and object-label association skills, β = 0.384, *t*(42) = 2.29, *p* = 0.027, Cohen’s *d* = 0.70. The control variables again did not reach significance as predictors of vocabulary change in the third regression model (Table 5C), as indicated by beta-coefficients for cognitive skill, SES and object-label association. Once again, toddlers’ looking to the statistically exposed “word” pairs performance in the experimental task accounted for a significant amount of variance in the vocabulary change from 18 to 24 months of age, with a moderate effect size, independent of cognitive skill, SES and object-label association skills, β = 0.370, *t*(42) = 2.19, *p* = 0.034, Cohen’s *d* = 0.67. Overall, both vocabulary production at 24 months old and vocabulary change were significantly predicted by looking to the statistically exposed “word” pairs at 18 months old, as indicated by significant *R*^2^ change resulting from adding difference scores to the regression model (see Tables 5A–C below).

**TABLE 5A–C |** Multiple regression analyses with looking to the statistically exposed word predicting vocabulary measures.

**TABLE 5A:** Summary of multiple regression predicting vocabulary at 18-mos.

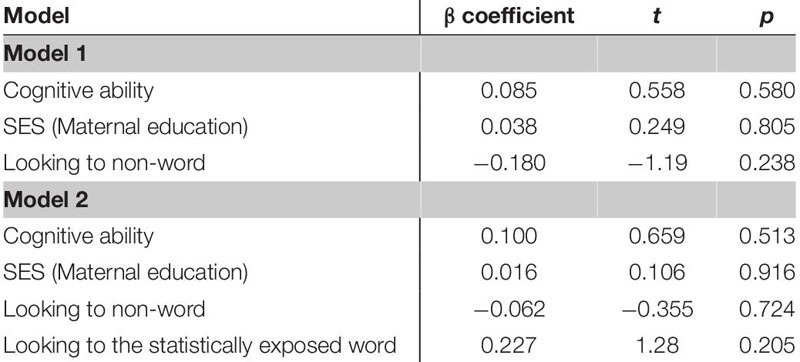

**N* = 47. *R*^2^ = 0.043 for Model 1; Δ*R*^2^ = 0.036 for Model 2 (*p* = 0.205); (one-tailed).*

**TABLE 5B:** Summary of multiple regression predicting vocabulary at 24-mos.

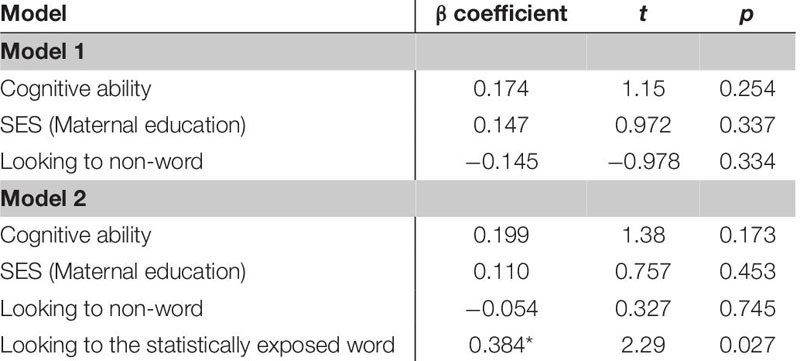

**N* = 47. *R*^2^ = 0.070 for Model 1; Δ*R*^2^ = 0.104 for Model 2 (*p* = 0.027). ^∗^<0.05 level (one-tailed).*

**TABLE 5C:** Summary of multiple regression predicting vocabulary change from 18 to 24-mos.

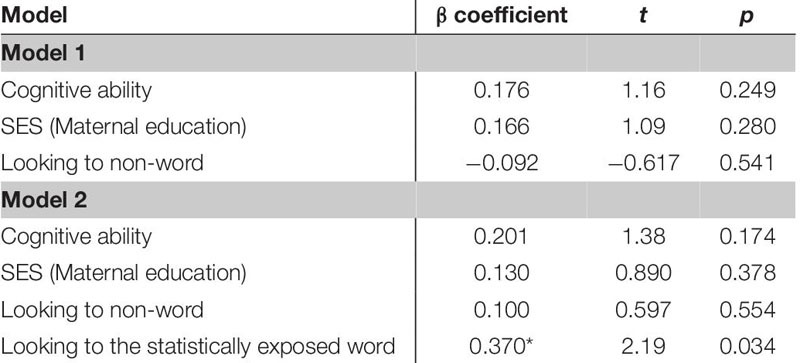

**N* = 47. *R*^2^ = 0.157 for Model 1; Δ*R*^2^ = 0.096 for Model 2 (*p* = 0.034). ^∗^<0.05 level (one-tailed).*

The next regression model included the control variables of cognitive skill, SES and simple object-label association did not significantly predict vocabulary at 18 months. After controlling for these variables, the relation between word learning difference scores on the experimental task and vocabulary at 18 months approached the threshold for significance, though the beta coefficient and Cohen’s d indicated a moderate size effect, β = 0.499, *t*(42) = 1.73, *p* = 0.091, Cohen’s *d* = 0.54 (see Table 5D). In the following regression, the control variables again did not reach significance as predictors of vocabulary at 24 months. However, the toddlers’ performance in the experimental task accounted for a significant amount of variance in their vocabulary at 24 months with a large effect size, independent of cognitive skill, SES and object-label association skills, β = 0.743, *t*(42) = 2.75, *p* = 0.009, Cohen’s *d* = 0.85 (see Table 5E). The control variables again did not reach significance as predictors of vocabulary change in the third regression model (see Table 5F), as indicated by beta-coefficients for cognitive skill, SES and object-label association. Once again, toddlers’ performance in the experimental task accounted for a significant amount of variance in the vocabulary change from 18 to 24 months of age, with a moderate-to-large effect size, independent of cognitive skill, SES and object-label association skills, β = 0.686, *t*(42) = 2.48, *p* = 0.017, cohen’s *d* = 0.77. Overall, both vocabulary production at 24 months old and vocabulary change were significantly predicted by looking difference scores at 18 months old, as indicated by significant *R*^2^ change resulting from adding difference scores to the regression model (see Tables 5D–F below).

**TABLE 5D–F |** Multiple regression analyses with looking difference scores predicting vocabulary measures.

**TABLE 5D:** Summary of multiple regression predicting vocabulary at 18-mos.

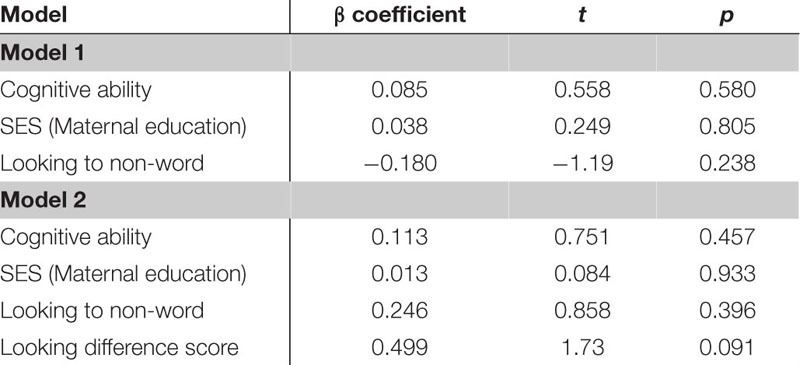

**N* = 47. *R*^2^ = 0.043 for Model 1; Δ*R*^2^ = 0.064 for Model 2 (*p* = 0.091); (one-tailed).*

**TABLE 5E:** Summary of multiple regression predicting vocabulary at 24-mos.

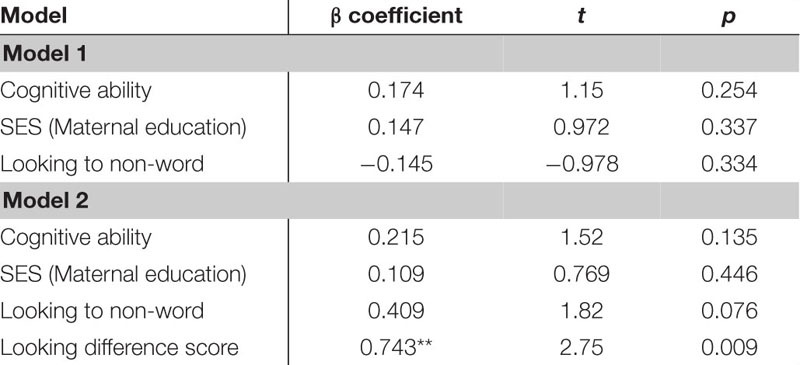

**N* = 47. *R*^2^ = 0.07 for Model 1; Δ*R*^2^ = 0.142 for Model 2 (*p* < 0.01). ^∗∗^<0.01 level (one-tailed).*

**TABLE 5F:** Summary of multiple regression predicting vocabulary change from 18 to 24-mos.

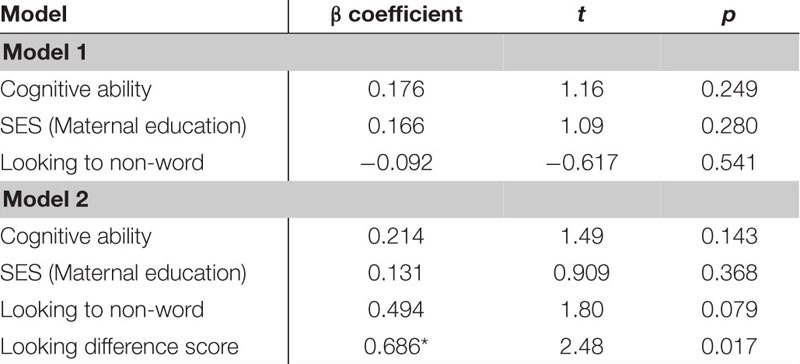

**N* = 47. *R*^2^ = 0.061 for Model 1; Δ*R*^2^ = 0.121 for Model 2 (*p* < 0.05). ^∗^<0.05 level (one-tailed).*

### *Post hoc* Analysis: Learning Patterns and Vocabulary

**FIGURE 4:**
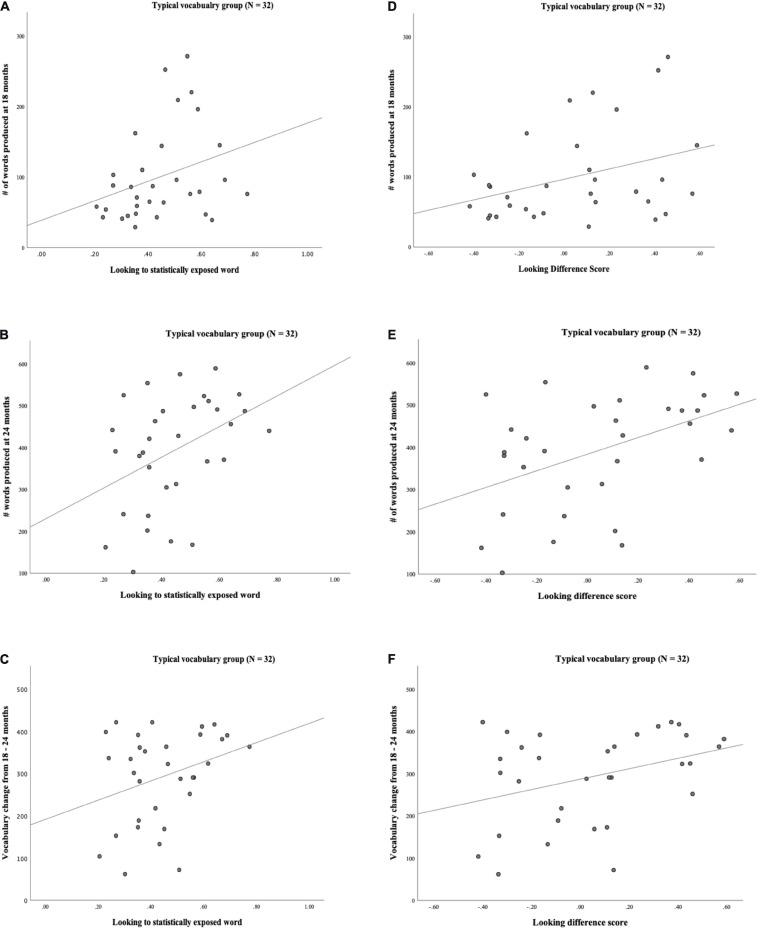
**(A)** Scatterplot of Looking to statistically exposed word and # words produced at 18 mos. for Typical vocabulary group (*N* = 32). (B) Scatterplot of Looking to statistically exposed word and # words produced at 24 mos. for Typical vocabulary group (*N* = 32). **(C)** Scatterplot of Looking to statistically exposed word and vocabulary change from 18 to 24 mos. for Typical vocabulary group (*N* = 32). **(D)** Scatterplot of Looking difference score and # words produced at 18 mos. for Typical vocabulary group (*N* = 32). **(E)** Scatterplot of Looking difference score and # words produced at 24 mos. for Typical vocabulary group (*N* = 32). **(F)** Scatterplot of Looking difference score and vocabulary change from 18 to 24 mos. for Typical vocabulary group (*N* = 32).

**FIGURE 5:**
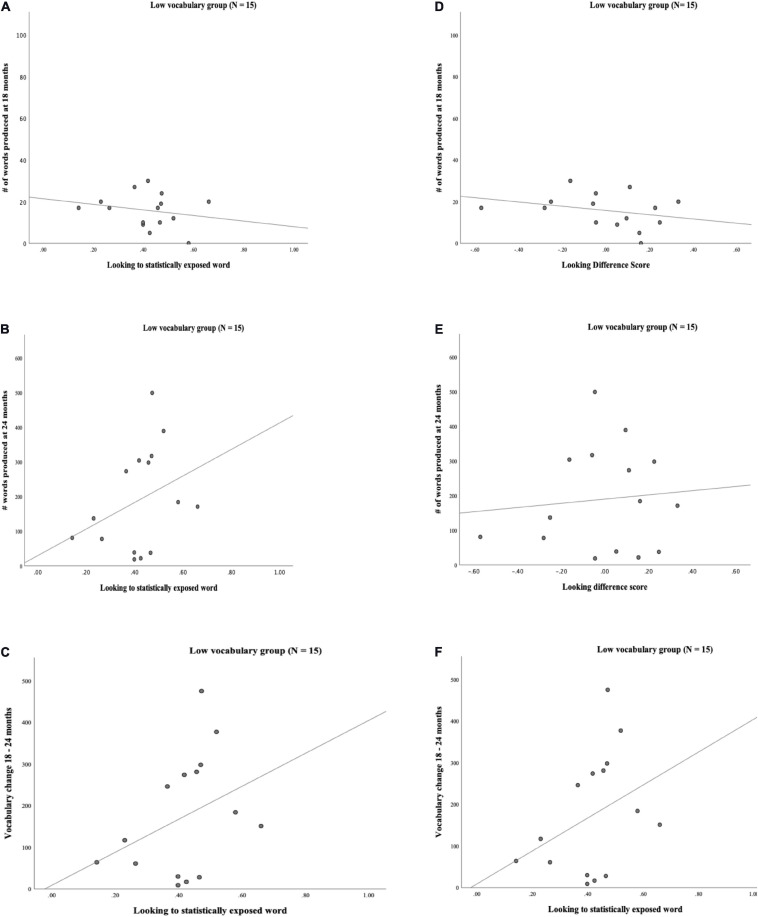
**(A)** Scatterplot of Looking to statistically exposed word and # words produced at 18 mos. for Low vocabulary group (*N* = 15). **(B)** Scatterplot of Looking to statistically exposed word and # words produced at 24 mos. for Low vocabulary group (*N* = 15). **(C)** Scatterplot of Looking to statistically exposed word and vocabulary change 18–24 mos. for Low vocabulary group (*N* = 15). **(D)** Scatterplot of Looking difference score and # words produced at 18 mos. for Low vocabulary group (*N* = 15). **(E)** Scatterplot of Looking difference score and # words produced at 24 mos. for Low vocabulary group (*N* = 15). **(F)** Scatterplot of Looking difference score and vocabulary change 18–24 mos. for Low vocabulary group (*N* = 15).

The extensive variability in the data was expected given the normal variability of word learning in the age range tested. This variability, however, led us to ask whether children who were reported to have low vocabulary would show substantially different patterns of word learning performance compared to children who did not have this designation. We followed precedent in other studies which designate <20^th^ percentile in productive vocabulary as “late-talking” (Fernald and Marchman, 2012). Fifteen of the 47 (32%) toddlers had scores at or below the 20^th^ percentile and 32 of the 47 (68%) toddlers had scores above the 20^th^ percentile. (Scatterplots of vocabulary and looking behaviors by vocabulary group are displayed in Figures 4A–F, 5A–F).

Several *post hoc* analyses were conducted including an analysis of the additional control of 18 month vocabulary to the regressions as well as examine the interaction of vocabulary group and looking behavior. The *post hoc* regression models included the control variables of cognitive skill, SES, simple object-label association and the added control of 18 month vocabulary. When examining the looking to the statistically exposed “word” pairs performance in the experimental task, 18 month vocabulary was significant in predicting vocabulary at 24 months in all 3 models. In model 2, both looking to the statistically exposed word and vocabulary group predicted 24 month vocabulary outcomes. In model 3 looking to the statistically exposed word was no longer a predictor and the interaction was non-significant (See Table 5G). In the *post hoc* regression predicting vocabulary change between 18 and 24 months model 1 only had one significant predictor of 18 month vocabulary, while in model 2 looking to the statistically exposed “word” pairs performance in the experimental task and vocabulary group were both significant predictors of vocabulary change. Model 3 no variables predicted the outcome variable of vocabulary change (see Table 5H). However, in the next analysis when examining the looking difference scores in model 1 the only significant predictor of vocabulary at 24 months is 18 month vocabulary. In model 2, 18 month vocabulary, looking to the statistically exposed word and vocabulary group each predicted 24 month vocabulary outcomes. In model 3 only 18 month vocabulary and vocabulary group was significant in predicting vocabulary at 24 months and the interaction was non-significant (see Table 5I). The final *post hoc* regression found 18 month vocabulary was only predictive of vocabulary change between 18 and 24 months in model 1, while model 2 the looking difference scores and vocabulary group were significant predictors, and model 3 only vocabulary group was significant in predicting vocabulary change (see Table 5J). Most interestingly, the toddlers’ looking difference score performance accounted for a significant amount of variance in their vocabulary change from 18 to 24 months with a moderate effect size, independent of cognitive skill, SES, object-label association skills, and 18 month vocabulary, β = 0.616, *t*(42) = 2.32, *p* = 0.025, Cohen’s *d* = 0.71 (see Table 5J).

**TABLE 5G–H |** *Post hoc* multiple regression analyses with looking to the statistically exposed word predicting vocabulary measures controlling for 18 month vocabulary.

**TABLE 5G:** *Post hoc* multiple regression predicting vocabulary at 24-mos.

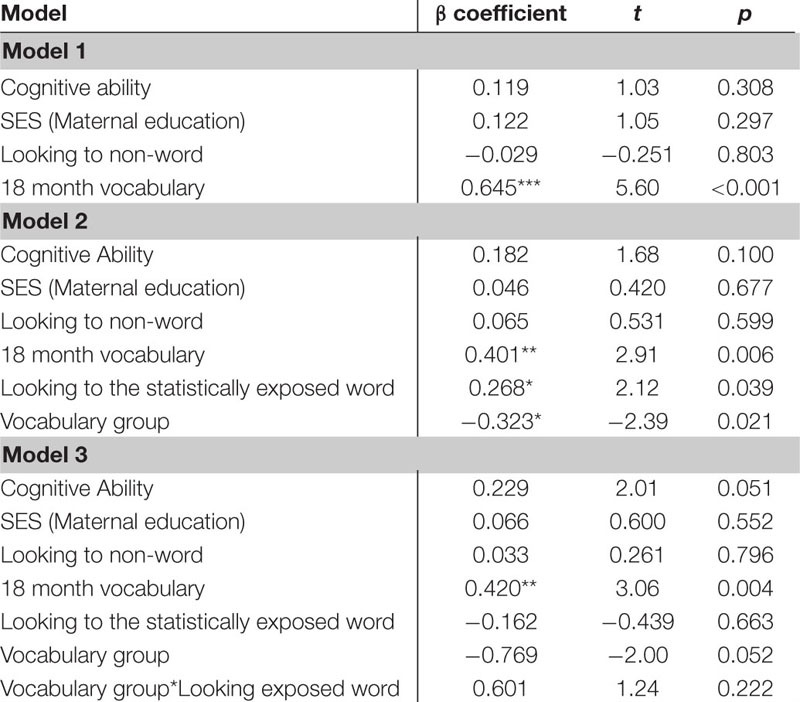

*N* = 47. *R*^2^ = 0.468 for Model 1 (*p* = 0.000); Δ*R*^2^ = 0.103 for Model 2 (*p* = 0.014); Δ*R*^2^ = 0.016 for Model 3 (*p* = 0.222) *≤0.05 level (one-tailed); **≤0.01 level (one-tailed); ***≤0.001 level (one-tailed).

**TABLE 5H:** *Post hoc* multiple regression predicting vocabulary change from 18 to 24-mos.

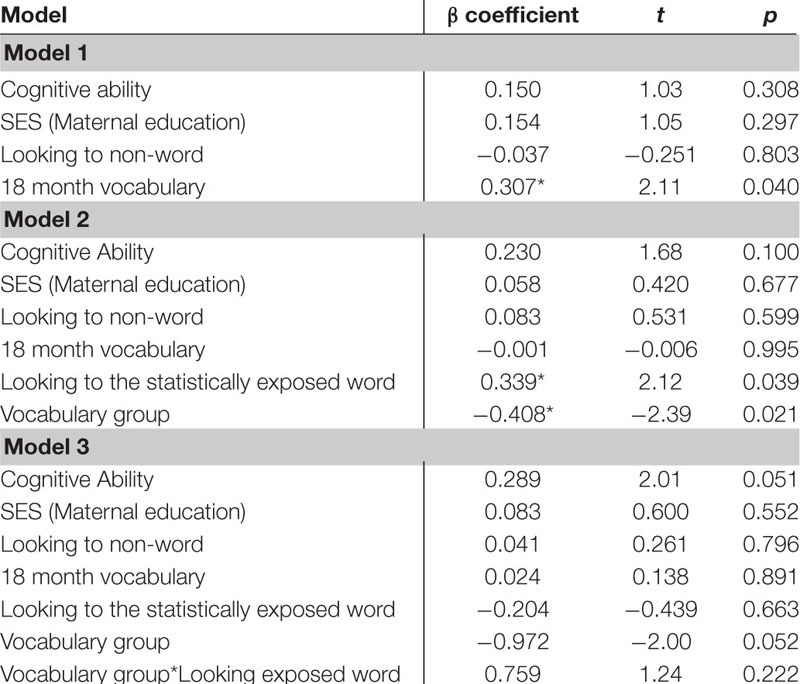

*N* = 47. *R*^2^ = 0.151 for Model 1 (p = 0.133); Δ*R*^2^ = 0.164 for Model 2 (p = 0.014); Δ*R*^2^ = 0.026 for Model 3 (p = 0.222) *≤0.05 level (one-tailed).

**TABLE 5I–J |** *Post hoc* multiple regression analyses with looking difference scores predicting vocabulary measures controlling for 18 month vocabulary.

**TABLE 5I:** *Post hoc* multiple regression predicting vocabulary at 24-mos.

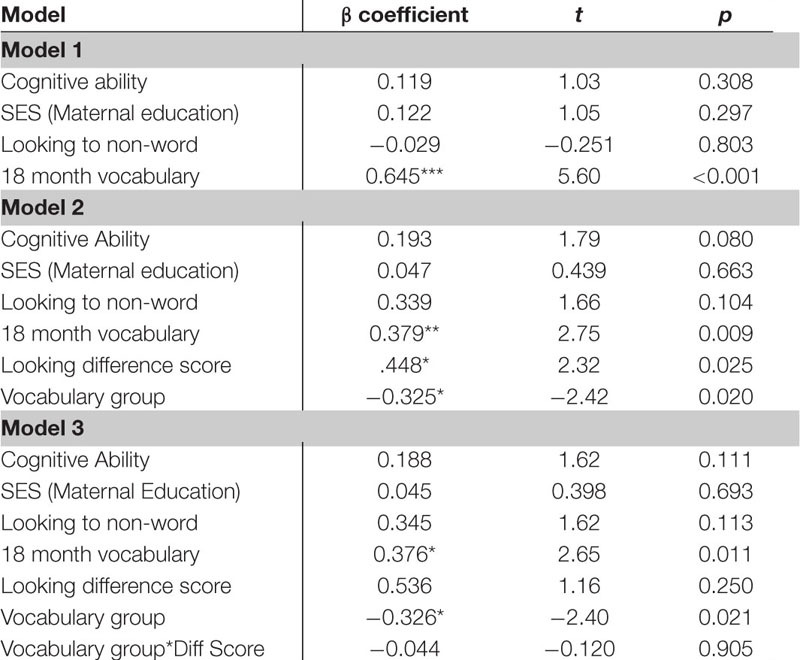

*N* = 47. *R*^2^ = 0.468 for Model 1 (p = 0.000); Δ*R*^2^ = 0.111 for Model 2 (p = 0.009); Δ*R*^2^ = 0.000 for Model 3 (p = 0.905); *≤0.05 level (1-tailed); **≤0.01 level (1-tailed).

**TABLE 5J:** *Post hoc* multiple regression predicting vocabulary change from 18 to 24-mos.

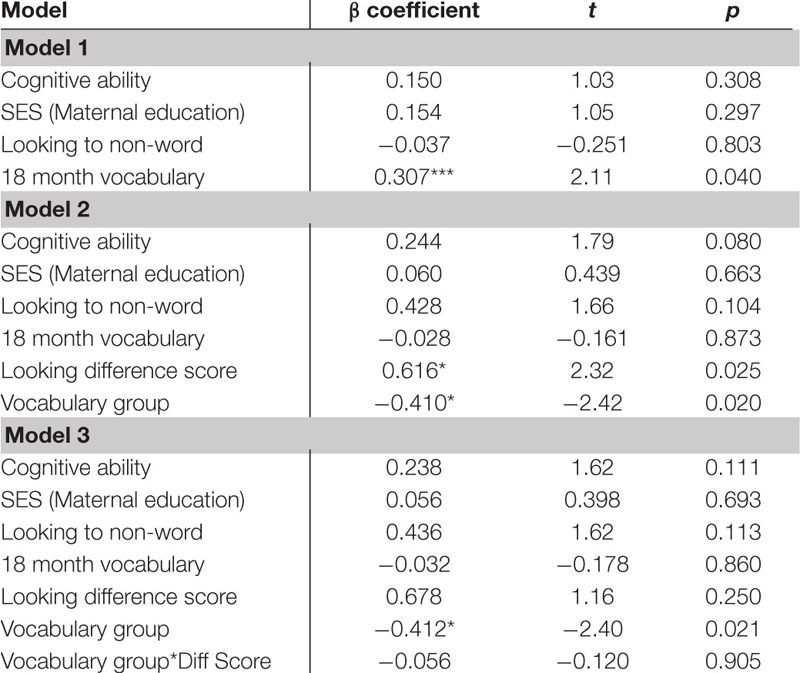

*N* = 47. *R*^2^ = 0.151 for Model 1 (p = 0.133); Δ*R*^2^ = 0.177 for Model 2 (p = 0.009); Δ*R*^2^ = 0.000 for Model 3 (p = 0.905); *≤0.05 level (1-tailed)

Next, we carried out a *post hoc* analysis that compared performance between “low vocabulary” toddlers who scored below the 20^th^ percentile on the MBCDI, and those who fell above this threshold. The descriptive statistics for each *post hoc* group suggested that the two groups had different patterns in looking behavior that did not rise above the level of statistical significance. As expected, the groups varied in vocabulary measures (see Table 6 for descriptive statistics). A *post hoc* correlation analysis suggests a significant positive relation between looking to statistically exposed “word” pairs and vocabulary measures at 18 months (*r* = 0.309, *p* = 0.043), 24 months (*r* = 0.404, *p* = 0.011), and vocabulary change (*r* = 0.310, *p* = 0.042). A similar pattern of results is seen for looking difference scores and vocabulary at 18 months (*r* = 0.348, *p* = 0.026), 24 months (*r* = 0.458, *p* = 0.004) and vocabulary change (*r* = 0.353, *p* = 0.024) for the higher, but not lower vocabulary group (see Table 7), suggesting that the differences in the group correlational and regression analyses above were largely driven by children who were above the low vocabulary threshold. While the *post hoc* analysis provide insight into vocabulary differences, this analysis should be interpreted with caution due to the small N, variation of group sizes and since we did not control for multiple comparisons.

**TABLE 6:** *Post hoc* descriptive statistics for toddlers split by vocabulary scores.

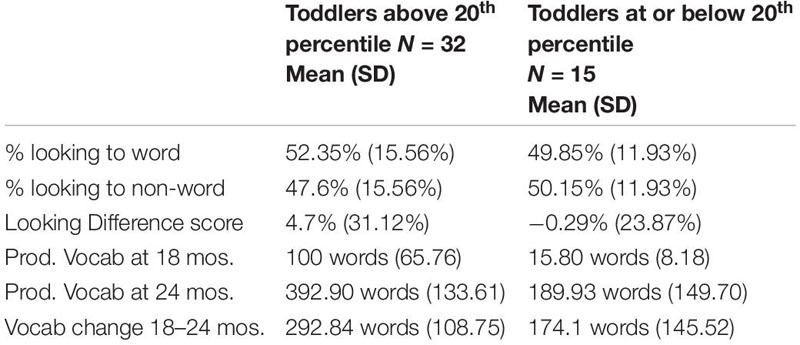

**TABLE 7:** Correlations between difference score and vocabulary measures by vocabulary groups.

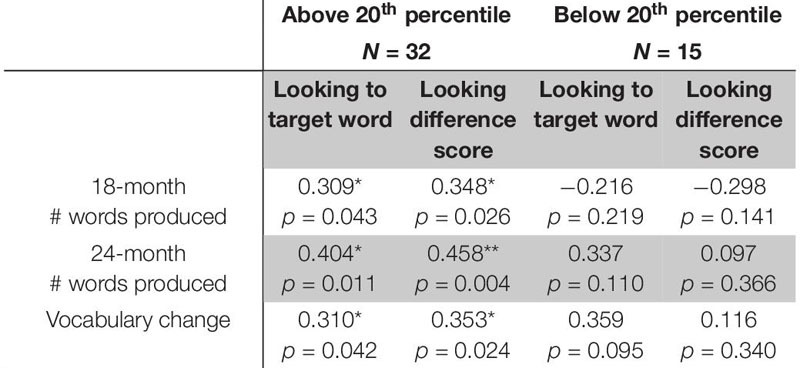

*^∗^<0.05 level (one-tailed). ^∗∗^<0.01 level (one-tailed).*

## Discussion

We predicted that if leveraging statistical information from a speech stream supports language development in typical young children, then the data should demonstrate a link between toddlers’ individual differences in linking previously exposed words and vocabulary outcomes. The predictions were generally supported by our results, with some exceptions and limitations. The findings support previous work (Graf Estes et al., 2007) and further affirm that the ability to decode the statistics of the sound sequences in language may influence vocabulary outcomes in toddlers. This is evidenced by the looking difference scores that predicted vocabulary outcomes. Previously, Graf Estes et al. (2007) found that prior exposure to a word form as a high-transitional probability sound sequence in an artificial language supported subsequent mapping of that word form to a label at 17 months of age. Our study builds on these seminal findings by exploring how variability in utilizing and leveraging statistical information from an artificial language supports not just direct object-label learning but also relate to toddlers’ vocabulary size and vocabulary change more generally.

Overall, the results suggest that toddlers showed a greater boost in learning as a function of this statistical pre-exposure to the word form also learned more words over time. Importantly, the results are not simply driven by children who are better at simply associating a label with an object, as indicated by the lack of significant association between the non-word condition and vocabulary size and change. In addition, the results were not driven by SES or cognitive skill, as our models included covariates of these factors as well. Instead, the findings suggest that vocabulary change was specifically related to leveraging statistical information and looking behavior. Importantly, because we measure language change prospectively, and find that the ability to use statistically learned words after exposure to the artificial statistical language is correlated with *later* language skills, our findings provide an additional indicator to the directionality of these results. Specifically, these findings suggest that a child’s ability to identify potential word forms from speech may drive subsequent word learning process and vocabulary change more generally.

While our findings suggest the statistical exposure may drive the children to look toward the statistically exposed word more often, we do need to highlight other possible interpretations given the large variability of looking patterns among the sample. Alternative explanations that consider young children’s ability to utilize statistical information is potentially not as robust as early studies suggest (Johnson and Tyler, 2010). We also acknowledge that some participants may show differences in looking behaviors that are reflective of above chance learning, while other participants may show the same difference in looking behaviors but below chance learning. Additionally, the observed patterns could be the result of or a combination of other processing abilities such as retaining memory for bigrams in the exposure language.

It is also important to note that our study design implemented several adaptations that vary from other experimental designs. These modifications were made to incorporate the current eye-tracking technology available at the time of testing. One of the advantages of using eye-tracking methodology, as opposed to traditional HPP paradigms, is it allows for examining aspects of the learning process. For example, by using a fixed number of trials, we controlled the object-label association phase to equally allow each participant the opportunity to hear and attend to the object-label pairs. We also used a gaze-contingent design to control the start point at each test trial. These important changes were essential to begin addressing the gaps in the literature.

As a group, toddlers showed no differences in word learning across the word and non-word conditions, however, there was a large amount of variability in the data. It is important to consider this variability with respect to the fact that our sample had cognitive scores within the normal range (*M* = 100.32, SD = 8.36, range = 85–115), no known language delay, impairment or other developmental diagnosis, and were only 18 months of age at the time of experimental testing. Even within this typically developing population, a range in looking behavior to both types of word targets was not unexpected. Importantly, this variation was not completely random: toddlers who showed a bigger boost in learning a novel object-label association from the statistically exposed condition also had better vocabulary skills at 18 and 24 months, and they also demonstrated greater vocabulary change between 18 and 24 months, while this relation between learning and vocabulary was not present for the non-words alone. Additionally, in the regression analyses the word learning measures as indicated by looking data are positive (B-coefficient), suggesting the measures are predicting toddlers who are better at learning the word (with high transitional probability) also demonstrated better vocabulary outcomes. Taken together, these results provide support for the idea that leveraging exposure to statistical information in a language support vocabulary change in general.

Few studies have examined (directly or indirectly) the link between statistical word learning and later language development in young toddlers. Our project used the Graf Estes et al. (2007) study as a foundation to explore a modified design to examine the link between statistical exposure, word learning, and extant vocabulary knowledge using a large sample of toddlers. Additionally, our design had an important control of testing two types of words to determine whether potential ties to vocabulary outcomes were simply a relation of general learning skills, or due to leveraging statistical information to support learning. The results of the current study suggest statistical exposure is an important factor contributing to linking potential word labels to objects and vocabulary skills in toddlers. In our current data, the significant effects of artificial language exposure are evident in vocabulary at 24 months old, but not earlier. While prior work suggests that statistical learning skills are important for language learning in school age children, the results of this study critically show a modest relation, in a large group of toddlers with a range of vocabulary abilities and, over time, independent of cognitive skill, SES and general object-label association skills. These findings have both theoretical and clinical implications for understanding language development and the risk for language learning delays.

Since factors of SES (as determined by maternal education) and general cognitive skills (Bayley scores) are not associated with the findings, we believe the results are tapping into general learning mechanisms that may play a role in lexical processing skills important for language learning. There is already existing literature on the importance of language-related processes such as processing speed and working memory in language development (e.g., Marchman and Fernald, 2008; Fernald and Marchman, 2012; Newbury et al., 2015; Peter et al., 2019). Our work adds to the growing knowledge (Hall et al., 2018; Lany et al., 2018; Tomas and Vissers, 2019) of how statistical learning skills among other important mechanisms may also be a critical language related process important for language development. Similarly to how some typical children may be faster or slower at processing information (Marchman and Fernald, 2008; Fernald and Marchman, 2012), vary in their memory skills (Newbury et al., 2015) and attention (Gomes et al., 2000; Tomas and Vissers, 2019) our results suggest that there may be aspects of individual differences within the realm of statistical learning abilities. More specifically, given recent findings that support infant statistical learning abilities relation to language processing (Lany et al., 2018), our research supports this account as we see toddlers’ abilities to utilize statistical regularities from fluent speech and map potential words to meaning at 18 months predicts vocabulary at 24 months suggesting it is supporting language development over time.

### Limitations

While these results are promising and suggest the ability to utilize statistical information may be an important factor to examine in hopes of better understanding vocabulary learning, our study does have limitations. It is clear that at the group level there were no significant differences in word and non-word label learning as well as a large amount of variability of performance in both looking behaviors and vocabulary skills contributed to our lack of significant group learning. Another potential limitation in our study is the measure of looking to both types of words is not necessarily independent. However, the multiple regression analyses controlled for learning of the non-word and thus, if toddlers were good at learning in both conditions this variance would be reflected in the non-word learning condition as well. The limitations above should be considered though, as we cautiously make our interpretations. Nevertheless, the findings of individual differences provide a useful first step in unraveling the complex, but important relation between language learning mechanisms and language ability.

## Conclusion

A child’s ability to track statistical information such as transitional probability and link a novel word to a novel picture is but one of many factors that support word learning. The field has identified a wide range of cognitive mechanisms that support early language development and word learning. For example, language-related cognitive processes such as speed of processing and working memory are important factors in children’s language development (e.g., Marchman and Fernald, 2008; Fernald and Marchman, 2012; Newbury et al., 2015; Peter et al., 2019). Similarly, other environmental factors can support learning as well, such as parental SES and linguistic input (Hart and Risley, 1995). There is still much yet to be known regarding how these mechanisms interact with statistical learning skills and, ultimately, word learning. Our research begins to answer some of these questions through the use of multiple regression models that include at least one prominent environmental factor – that of SES – as well as a broad measure of non-verbal cognitive skill (Bayley Cognitive standard score). Our data suggests that the relation between skills in leveraging statistical information and word learning persist even when controlling for these other environmental and cognitive factors. This finding supports the need for further and more comprehensive investigations that disentangle precisely how, and when, multiple factors that support language learning interact and relate to longer-term language outcomes and assess a broad range of domain-general factors that support early language learning.

Examining lexical and cognitive processing skills – that is, how individuals learn, process, and think about characteristics of words – in a large sample of toddlers with a range of abilities may better inform understanding of individual differences in typical language development. More importantly, it lays the foundation to start determining what may be considered normal variability for statistical word learning abilities – one of several important mechanisms for word learning. Similar to Fernald and Marchman (2012) findings that individual differences in processing speed in infancy are related to real language outcomes, such as vocabulary size and growth trajectories, statistical word learning skills may be an important factor to examine within the statistical learning account of language acquisition. A better understanding of statistical word learning mechanisms, its relation to word learning and to vocabulary, and later language outcomes may help researchers and clinicians explore which characteristics may be important indicators for toddlers at risk for later language disorders (Thiessen et al., 2013; Erickson and Thiessen, 2015; Saffran, 2018). Future efforts will examine longitudinal data linking statistical word learning abilities in toddlers to later language outcomes in young children with language delays and disorders.

## Author’s Note

Data was collected at the University of California, San Diego, when the EE was completing graduate studies and the AB was completing a postdoctoral position. Current institutional affiliations are reflected above. JEl passed away in 2018 during preparation of the current manuscript.

## Data Availability Statement

The datasets presented in this article are not readily available due to IRB restrictions. Requests to access the datasets should be directed to EE, eellisr@calstatela.edu.

## Ethics Statement

The studies involving human participants were reviewed and approved by the University of California, San Diego IRB. Written informed consent to participate in this study was provided by the participants’ legal guardian/next of kin.

## Author Contributions

All authors listed have made a substantial, direct and intellectual contribution to the work, and approved it for publication.

## Conflict of Interest

The authors declare that the research was conducted in the absence of any commercial or financial relationships that could be construed as a potential conflict of interest.

## Footnotes

1 http://www.tarrlab.org/

2 Sample included average of 84.37% looking across target and distractor trials (min. = 58.03%).
